# Sexual health in long-term breast cancer survivors: a comparison with female population controls from the HUNT study

**DOI:** 10.1007/s10549-023-07021-y

**Published:** 2023-07-25

**Authors:** Solveig K. Smedsland, Kathrine F. Vandraas, Ragnhild S. Falk, Julie Horn, Randi J. Reidunsdatter, Cecilie E. Kiserud, Alv A. Dahl, Mette Brekke, Kristin V. Reinertsen

**Affiliations:** 1 National Advisory Unit for Late Effects After Cancer Treatment, Department of Oncology, Oslo University Hospital, Postboks 4950 Nydalen, 0424 Oslo, Norway; 2 Department of Community Medicine and Global Health, Institute of Health and Society, University of Oslo, Oslo, Norway; 3 Research Support Services, Oslo Centre for Biostatistics and Epidemiology, Oslo University Hospital, Oslo, Norway; 4 Department of Public Health and Nursing, Norwegian University of Science and Technology, Trondheim, Norway; 5 Department of Obstetrics and Gynecology, Levanger Hospital, Nord-Trøndelag Hospital Trust, Levanger, Norway; 6 Department of Circulation and Medical Imaging, Norwegian University of Science and Technology, Trondheim, Norway; 7 Department of General Practice, Institute of Health and Society, University of Oslo, Oslo, Norway

**Keywords:** Breast cancer survivors, Sexual health, Sexual functioning, Sexual enjoyment, Sexual discomfort

## Abstract

**Purpose:**

Sexual health is an important aspect of quality of life. Knowledge concerning sexual health in long-term breast cancer survivors (BCSs) is limited. This study compared sexual health in BCSs 8 years after diagnosis with similarly aged controls and examined the impact of menopausal status at diagnosis and systemic breast cancer treatments on sexual health.

**Methods:**

Women aged 20–65 years when diagnosed with stage I–III breast cancer in 2011–2012 were identified by the Cancer Registry of Norway (*n* = 2803) and invited to participate in a nationwide survey. Controls were women from the Trøndelag Health Study (HUNT4). Sexual functioning and sexual enjoyment were measured by the EORTC QLQ-BR23 subscales scored from 0 to 100, and sexual discomfort by the Sexual Activity Questionnaire scored from 0 to 6. Linear regression analyses with adjustments for sociodemographic and health-related variables were performed to compare groups. Differences of ≥ 10% of range score were considered clinically significant.

**Results:**

The study samples consisted of 1241 BCSs and 17,751 controls. Sexual enjoyment was poorer (B − 13.1, 95%CI − 15.0, − 11.2) and discomfort higher (B 0.9, 95%CI 0.8, 1.0) among BCSs compared to controls, and larger differences were evident between premenopausal BCSs and controls (B − 17.3, 95%CI − 19.6, − 14.9 and B 1.2, 95%CI 1.0, 1.3, respectively). BCSs treated with both endocrine- and chemotherapy had lower sexual functioning (B − 11.9, 95%CI − 13.8, − 10.1), poorer sexual enjoyment (B − 18.1, 95%CI − 20.7, − 15.5), and more sexual discomfort (B 1.4, 95% 1.3, 1.6) than controls.

**Conclusion:**

Sexual health impairments are more common in BCSs 8 years after diagnosis compared to similar aged population controls. During follow-up, attention to such impairments, especially among women diagnosed at premenopausal age and treated with heavy systemic treatment, is warranted.

**Supplementary Information:**

The online version contains supplementary material available at 10.1007/s10549-023-07021-y.

## Introduction

Breast cancer (BC) is the most frequent female cancer globally with an estimated 2.3 million new cases in 2020 [1]. The 5-year relative survival is steadily increasing and has surpassed 90 percent in several western countries [2, 3]. Consequently, the population of BC survivors (BCSs) is growing, making their quality of life a key outcome measure besides to survival [4, 5].

Sexual health, defined as a state of physical, emotional, mental and social well-being related to sexuality [6], is an important aspect of quality of life [7, 8]. Treatment for stage I–III BC is often intense including combinations of surgery, radiotherapy, and adjuvant systemic therapies (endocrine therapy, chemotherapy, and targeted therapy), which may have a negative impact on sexual health [8–10]. Studies have focused on various aspects of sexual health, like sexual activity, interest, enjoyment and discomfort, and reported that sexual health impairments are prevalent among BCSs both during treatment and during the first years after diagnosis [11–15]. However, it remains unclear if these sexual health impairments persist long-term (> 5 years after diagnosis), as data from this part of survivorship are scarce and results conflicting [16–19]. A recent study from our research group found that adjuvant treatment with aromatase inhibitors, extended endocrine treatment and adjuvant chemotherapy were associated with sexual health impairments also in long-term BCSs [20]. As in several prior studies, a limitation with our study was the lack of a general population control sample.

In the general population, increasing age is associated with reduced sexual activity and increasing sexual complaints [21, 22]. In BC populations, also women at a younger age seem to be at high-risk of sexual health impairments [23, 24]. This may be due to troublesome estrogen deprivation symptoms caused by systemic adjuvant BC therapies. Several studies report that younger BCSs with chemotherapy-induced premature menopause are at special risk of such impairments the first years after diagnosis [13, 25]. Endocrine therapies also demonstrate a clear negative effect on sexual health the first 5 years after diagnosis [8, 11, 26, 27]. For most BC patients, the recommended duration of endocrine therapy is 5 years, but extended therapy for up to 10 years is an option for high-risk patients [28]. Consequently, a subgroup of patients may struggle with estrogen deprivation symptoms and sexual health impairments during the first decade after their BC diagnosis. This subgroup includes women of childbearing age where unimpaired sexual health may be especially important.

Given the scarce and inconclusive evidence of sexual health in long-term BCSs, there is a need for large-scale studies comparing sexual health among long-term BCSs to that of similarly aged population controls.

The aims of the present study were: (1) to compare sexual health among long-term BCSs to that of similarly aged female controls from a population-based sample, (2) to assess the impact of systemic BC treatments on sexual health, and (3) to examine if menopausal status at BC diagnosis influences sexual health.

## Material and method

### Breast cancer survivors

This study is part of the **S**urvivorship-**w**ork-s**e**xual-h**e**al**t**h (SWEET) study, a cross-sectional survey examining work life and sexual health among Norwegian long-term BCSs. All women diagnosed with BC stage I–III in 2011 or 2012 at the age of 20–65 years were identified by the Cancer Registry of Norway (CRN). CRN is based on mandatory reporting, and has close- to complete registration of all cancer cases in Norway [29]. To be included in SWEET, women had to be free of pre- or post- malignancies (except non-melanoma skin cancer and ductal carcinoma in situ). Invitation was mailed to 2803 BCSs in December 2019 and one reminder was sent to non-responders (*n* = 1684) in February 2020. The questionnaire was returned by 1361 BCSs (49% response rate). We excluded BCSs with either missing consent or self-reported BC recurrence (*n* = 6) and BCSs with missing data on sexual activity (*n* = 114), resulting in a final sample of 1241 BCSs. Characteristics of non-responders and attrition analysis of SWEET have previously been reported [20]. BCSs with missing data on sexual activity were older, fewer lived with a partner, and they had shorter education compared to the included BCSs (data not shown).

### Population controls

SWEET participants were compared to similarly aged women participating in the fourth survey of the population-based Trøndelag Health Study (HUNT4) [30]. HUNT4 invited all adults in the Nord-Trøndelag region aged 20 years and above to an extensive health assessment during 2017–2019. Participants responded to questionnaires and attended clinical examinations. In total, 18,782 women in the age group relevant for this study (30–74 years) participated (52% of those invited). Due to missing data on sexual activity, 1031 women were excluded, resulting in a control group of 17,751 women. Controls with missing data on sexual activity were older, fewer lived with a partner, had shorter education, more somatic co-morbidity and less mental co-morbidity compared to the included controls (data not shown).

### Sexual health measures in SWEET and HUNT4

The sexual health outcomes explored in this study were sexual functioning, sexual enjoyment, and sexual discomfort.

The European Organization for Research and Treatment of Cancer Quality of Life Questionnaire BC-specific module (EORTC-BR23) [31] includes three items exploring sexual health during the past 4 weeks. Two items concerning sexual interest and activity assess sexual functioning, and one item assesses sexual enjoyment among those who are sexually active. Responses were rated from 1 (not at all) to 4 (very much) and transformed to a 0–100 scale according to the scoring manual [32]. Higher scores indicate better sexual functioning and sexual enjoyment. Sexually active women were defined as being sexually active (with or without intercourse) during the past 4 weeks.

Sexually active women also responded to two items from The Sexual Activity Questionnaire [33], assessing sexual discomfort during the past 4 weeks. Responses were rated from 0 (not at all) to 3 (very much) and summarized, yielding a sum score ranging from 0 to 6, where a higher score correspond to more sexual discomfort.

### Covariates

Both in SWEET and HUNT4 sociodemographic information included age at survey, self-reported living arrangements (partner or not) and length of education (≤ 12 years/ > 12 years).

Somatic comorbidities included a self-reported history of heart-, pulmonary-, thyroid-, kidney-, or rheumatic disease, cerebral stroke and/or diabetes, and were categorized into three; no comorbid condition, 1 or ≥ 2 comorbid conditions.

Mental co-morbidity was assessed by one question regarding help seeking for mental health problems (Yes/No).

Sleep problems were present when participants reported more than three episodes per week of difficulty falling asleep and/or waking up too early without going back to sleep (Yes/No). Recall time was the past 3 months [34].

Body mass index (BMI) was calculated from height and weight, self-reported in SWEET and from standardized measurements at the field stations in HUNT4.

### Menopausal status

As information on menopausal status at diagnosis was unavailable in SWEET, we used age as a proxy. The estimated mean age of menopause in Norwegian women is 52.7 years [35]. In order to avoid misclassification of premenopausal women, postmenopausal status was defined as being ≥ 55 years.

### Cancer-related information

In SWEET, age at diagnosis, BC stage, and surgical treatment were retrieved from the CRN. Information on adjuvant radiotherapy and on systemic adjuvant therapies was self-reported. Systemic treatment was categorized into no systemic treatment, endocrine therapy only, chemotherapy only, and endocrine- and chemotherapy.

Information on prior or present cancer among participants in HUNT4 was self-reported.

### Statistical analysis

Descriptive statistics are presented as frequencies and proportions for categorical data, where missing data are given as a separate category. Continuous data are presented with mean and standard deviation.

The three outcome variables sexual functioning, sexual enjoyment and sexual discomfort, were compared between BCSs and controls using linear regression analyses. Two models were performed, the first with adjustments for the sociodemographic variables (age at survey, living with partner or not, education), and the second with additional adjustments for the health-related variables (somatic co-morbidity, mental co-morbidity, BMI and sleep problems). Further, the analyses were stratified by age to study the impact of pre-/postmenopausal age at diagnosis. When exploring the impact of systemic BC therapies on the sexual outcome variables, we only adjusted for the sociodemographic variables, as the health-related covariates may be considered as mediators. Results are presented as beta coefficients (B) with 95% confidence intervals (CI).

A clinically significant difference between the groups was defined as a mean score difference of ≥ 10% of range scores [36, 37], i.e., ≥ 10 scale points for sexual functioning and enjoyment and ≥ 0.6 for sexual discomfort.

Due to missing data in several of the covariates included in the analyses, multiple imputations procedures were performed. These analyses yielded similar results compared to the main results, and were therefore not presented.

Analyses were performed using IBM SPSS statistics version 28.0 (Armonk, NY) and Stata version 17 (StataCorp LLC, College Station, TX).

## Results

### Characteristics of BCSs and controls

Mean age of BCSs was 59 years at survey, and 8 years had passed since diagnosis. Most BCSs had been treated for BC stage I or II (80%) with breast conserving therapy (58%), radiotherapy (80%) and/or systemic treatment with both chemo- and endocrine therapy (54%). Sixty-two percent were premenopausal when diagnosed with BC. BCSs premenopausal at diagnosis had more advanced disease and higher treatment burden compared to BCSs who were postmenopausal at diagnosis (*p*-values < 0.001).

The controls were younger (mean age of 54 years) than the BCSs, and 7% reported prior or present cancer. BCSs and controls had similar prevalence of somatic co-morbidity, but BCSs had more mental co-morbidity, more sleep problems and lower BMI compared to controls (all *p*-values < 0.001). (Table 1).

**Table 1:** Characteristics of breast cancer survivors (SWEET) and population controls (HUNT4)

	SWEET (*n* = 1241)			HUNT4 (*n* = 17,751)	*p*-value
Sociodemographic variables					
Age at survey, mean (SD)	59.4 (8.6)			53.9 (12.0)	< 0.001
Living with a partner, *n* (%)	931 (75.0)			14,121 (79.6)	< 0.001
Education > 12 years, *n* (%)	658 (53.0)			8406 (47.4)	< 0.001
*Missing*	*11 (0.9)*			*56 (0.3)*	*
Health variables					
Somatic co-morbidity^a^, *n* (%)					0.29
No condition	753 (60.7)			10,730 (60.4)	
1 condition	330 (26.6)			4284 (24.1)	
≥ 2 or more conditions	101 (8.1)			1286 (7.2)	
*Missing*	*57 (4.6)*			1451 (8.2)	
Mental co-morbidity, *n* (%)	352 (28.4)			3775 (21.3)	< 0.001
*Missing*	*25 (2.0)*			*550 (3.1)*	
Body mass index, (kg/m^2^), mean (SD)	26.2 (4.4)			27.2 (5.0)	< 0.001
*Missing, n (%)*	*18 (1.5)*			68 (0.4)	
Sleep problems, *n* (%)	547 (44.1)			3830 (21.6)	< 0.001
*Missing*	*15 (1.2)*			*482 (2.7)*	

Cancer-related variables	In total (*n* = 1241)	Premenopausal at diagnosis^b^ (*n* = 769)	Postmenopausal at diagnosis^c^ (*n* = 472)		
Age at diagnosis, mean (SD)	51.4 (8.6)	46.1 (6.3)	60.0 (3.1)	−	−
Time since diagnosis, mean (SD)	8.0 (0.7)	8.0 (0.7)	8.1 (0.7)	−	−
Stage, *n* (%)					
I	541 (43.6)	276 (35.9)	265 (56.1)	−	−
II	450 (36.3)	309 (40.2)	141 (29.9)	−	−
III	103 (8.3)	69 (9.0)	34 (7.2)	−	−
*Missing*	*147 (11.8)*	*115 (15.0)*	*32 (6.8)*		
Breast conserving surgery, *n* (%)	718 (57.9)	381 (49.5)	337 (71.4)	−	−
Chemotherapy, *n* (%)	867 (69.9)	653 (84.9)	214 (45.3)	−	−
Radiotherapy, *n* (%)	994 (80.1)	601 (78.2)	393 (83.3)	−	−
Ever use of endocrine therapy (ET), *n* (%)	827 (66.7)	549 (71.4)	278 (58.9)	−	−
Current use of ET, *n* (%)	289 (23.3)	264 (34.3)	25 (5.3)	−	−
*Missing*	*34 (2.7)*	*11 (1.4)*	*23 (4.9)*		
Systemic treatment burden, n (%)					
No systemic treatment	211 (17.0)	81 (10.5)	130 (27.5)	−	−
ET only	153 (12.3)	31 (4.0)	122 (25.8)	−	−
Chemotherapy^d^ only	200 (16.1)	139 (18.1)	61 (13.0)	−	−
Chemotherapy^d^ and ET	667 (53.8)	514 (66.8)	153 (32.4)	−	−
*Missing*	*10 (0.8)*	*4 (0.5)*	*6 (1.3)*		

^a^Heart, pulmonary, thyroid, kidney, rheumatic disease, cerebral stroke, diabetes,

^b^Breast cancer survivors < 55 years at diagnosis,

^c^Breast cancer survivors ≥ 55 years at diagnosis,

^d^Including breast cancer survivors treated with trastuzumab

*SD* standard deviation

*Statistically significant*: *p* < 0.05

### Comparison of sexual health between all BCSs and controls

**Fig. 1:**
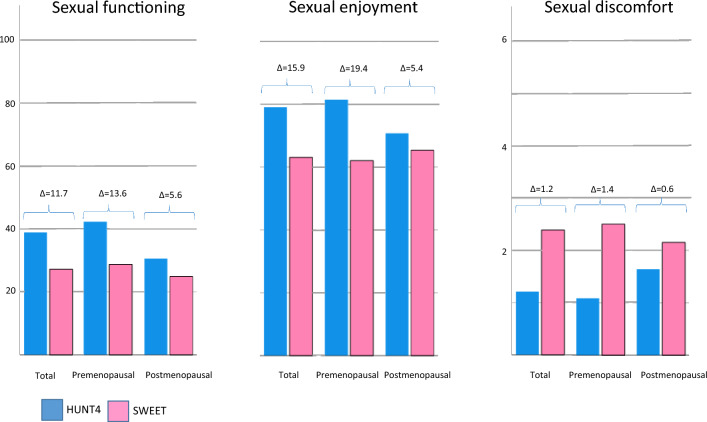
Sexual health in long-term breast cancer survivors (SWEET) and population controls (HUNT4)

All sexual health outcomes were poorer among BCSs than among controls (unadjusted mean scores for sexual functioning (27.2 vs 38.9), sexual enjoyment (63.1 vs 79.0) and sexual discomfort (2.4 vs 1.2) (Fig. 1).

When adjusting for sociodemographic variables, the BCSs had clinically significant lower sexual enjoyment (B − 14.4, 95%CI − 16.4, − 12.5) and more discomfort (B 1.0, 95%CI 0.9, 1.1) than controls. These differences slightly decreased, but remained clinically significant when additionally adjusting for health-related variables. However, for sexual functioning we found no clinically significant difference between BCSs and controls in the adjusted analyses (Table 2).

**Table 2:** Comparison of sexual health between breast cancer survivors (SWEET) and population controls (HUNT4) in total samples and separated by menopausal status

	Sexual functioning^a^		Sexual enjoyment^b^		Sexual discomfort^c^	
	Beta	95% CI	Beta	95% CI	Beta	95% CI
All age groups						
HUNT4	Ref	−	Ref	−	Ref	−
SWEET^d^	− 8.7	− 10.1, − 7.3	**− 14.4**	**− 16.4, − 12.5**	**1.0**	**0.9, 1.1**
HUNT	Ref	−	Ref	−	Ref	−
SWEET^e^	− 7.7	− 9.1, − 6.3	**− 13.1**	**− 15.0, − 11.2**	**0.9**	**0.8, 1.0**
Premenopausal status^f^						
HUNT4	Ref	−	Ref	−	Ref	−
SWEET^−d^	**− 11.0**	**− 12.8, − 9.2**	**− 18.9**	**− 21.2, − 16.6**	**1.3**	**1.2, 1.4**
HUNT4	Ref	−	Ref	−	Ref	−
SWEET^e^	− 9.7	− 11.5, − 7.9	**− 17.3**	**− 19.6, − 14.9**	**1.2**	**1.0, 1.3**
Postmenopausal status^g^						
HUNT4	Ref	−	Ref	−	Ref	−
SWEET^d^	− 5.3	− 7.6, − 2.9	− 5.7	− 9.3, − 2.2	0.4	0.2, 0.7
HUNT4	Ref	–	Ref	−	Ref	−
SWEET^e^	− 5.0	− 7.4, − 2.7	− 5.1	− 8.6, − 1.5	0.4	0.1, 0.6

Linear regression with sexual health measures as outcome variables

^a^Scale 0–100 (EORTC-BR23)

^b^Scale 0–100 (EORTC-BR23) among sexually active women

^c^Scale 0–6 (Sexual Activity Questionnaire) among sexually active women

^d^Adjusted for age at survey, living with a partner or not, education > / ≤ 12 years

^e^Adjusted for age at survey, living with a partner or not, education > / ≤ 12 years, somatic co-morbidity (no, 1, ≥ 2), mental co-morbidity (yes/no), body mass index, sleep problems (yes/no)

^f^Breast cancer survivors (BCSs) premenopausal at diagnosis (< 55 years) and similar aged controls

^g^BCSs postmenopausal at diagnosis (≥ 55 years) and similar aged controls

*CI* confidence interval

Bold: Clinically significant findings

The complete linear regression model with estimates is shown in Supplementary Table 1.

### Comparisons of sexual health between BCSs according to systemic treatment burden and controls

BCSs treated with both endocrine- and chemotherapy had clinically significant lower sexual functioning (B − 11.9, 95%CI − 13.8, − 10.1), lower sexual enjoyment (B − 18.1, 95% − 20.7, − 15.5), and more sexual discomfort (B 1.4, 95%CI 1.3, 1.6) compared to controls. These differences were smaller when comparing BCSs treated with endocrine- or chemotherapy only with controls, and were not clinically significant for sexual functioning. There were no clinically significant differences in any sexual health outcomes when comparing BCSs treated without systemic therapies and controls (Table 3).

**Table 3:** Comparison of sexual health between breast cancer survivors (SWEET) according to systemic treatment burden and population controls (HUNT4) in total samples and separated by menopausal status

	Sexual functioning^a^		Sexual enjoyment^b^		Sexual discomfort^c^	
	Beta	95% CI	Beta	95% CI	Beta	95% CI
All age groups						
HUNT4	Ref	–	Ref	–	Ref	–
SWEET – no systemic treatment	− 2.9	− 5.9, 0.7	− 6.4	− 10.9, − 1.8	0.3	0.04, 0.6
SWEET – ET only	− 5.8	− 9.6, − 1.9	**− 11.0**	**− 16.2, − 5.7**	**0.7**	**0.4, 1.0**
SWEET – chemo^d^ only	− 8.2	− 11.6, − 4.9	**− 12.6**	**−17.0, −8.2**	**0.8**	**0.5, 1.0**
SWEET – chemo^d^ + ET	**− 11.9**	**− 13.8, − 10.1**	**− 18.1**	**− 20.7, − 15.5**	**1.4**	**1.3, 1.6**
Premenopausal status^e^						
HUNT4	Ref	–	Ref	–	Ref	–
*SWEET – no systemic treatment*	*−*	*−*	*−*	*−*	−	−
*SWEET – ET only*	*−*	*−*	*−*	*−*	−	−
SWEET – chemo^d^ only	− 9.0	− 13.0, − 5.0	**− 15.8**	**− 20.8, − 10.8**	**0.9**	**0.6, 1.2**
SWEET – chemo^d^ + ET	**− 13.1**	**− 15.2, − 11.0**	**− 20.3**	**− 23.1, − 17.5**	**1.5**	**1.4, 1.7**
Postmenopausal status^f^						
HUNT4	Ref	−	Ref	−	Ref	−
SWEET – no systemic treatment	− 0.3	− 4.6, 3.9	0.8	− 5.5, 7.1	− 0.1	− 0.5, 0.3
SWEET – ET only	− 6.3	− 10.6, − 2.0	− 9.4	− 15.7, − 3.0	**0.6**	**0.2, 1.0**
* SWEET – chemo*^*d*^* only*	−	−	−	−	−	−
SWEET – chemo^d^ + ET	**− 10.0**	**− 13.9, − 6.2**	**− 12.0**	**− 18.2, − 5.8**	**1.0**	**0.6, 1.4**

Linear regression with sexual health measures as outcome variables. Adjusted for age at survey, living with a partner or not, education > / ≤ 12 years

^a^Scale 0–100 (EORTC-BR23)

^b^Scale 0–100 (EORTC-BR23) among sexually active women

^c^Scale 0–6 (Sexual Activity Questionnaire) among sexually active women

^d^Including BCSs treated with trastuzumab

^e^Breast cancer survivors (BCSs) premenopausal at diagnosis (< 55 years) compared to similar aged controls

^f^BCSs postmenopausal at diagnosis (≥ 55 years) compared to similar aged controls

*CI* confidence interval, *ET* endocrine therapy

Italics: Treatment groups with too small *n* to present valid statistical analyses

Bold: Clinically significant findings

### Stratified analyses according to age at diagnosis

The differences in unadjusted sexual health outcomes between BCSs and controls were most pronounced in the premenopausal group (Fig. 1).

BCSs who were premenopausal at diagnosis, had clinically significant lower sexual functioning (B − 11.0, 95%CI − 12.8, − 9.2), lower enjoyment (B − 18.9, 95%CI − 21.2, − 16.6) and more discomfort (B 1.3, 95%CI 1.2, 1.4) compared to similar aged controls after adjusting for sociodemographic variables. When additionally adjusting for health-related variables, sexual enjoyment and discomfort remained significantly worse among BCSs than among controls. There were no clinically significant differences in any of the sexual health outcomes when comparing BCSs diagnosed at postmenopausal age with similar aged controls (Table 2).

Separating BCSs treated with both endocrine- and chemotherapy in being pre-and postmenopausal at diagnosis, all sexual health outcomes were poorer among BCSs compared to controls. The differences observed were most pronounced in the premenopausal group (Table 3).

## Discussion

Eight years after diagnosis, BCSs had lower sexual enjoyment and more sexual discomfort compared to similarly aged controls. Sexual health impairments were most pronounced in BCSs who were premenopausal at diagnosis, and in BCSs with heavy systemic treatment burden.

Comparisons between BCSs and similar aged population controls are crucial to differentiate age-related changes in sexual health from those associated with BC diagnosis and treatment. However, only few such comparative studies have been performed. Dorval et al. [19] and Soldera et al. [18] reported no differences in sexual activity between long-term BCSs and controls. Their results partly correspond to our finding of no difference in sexual functioning between BCSs and controls in the adjusted analyses. In line with our results showing poorer sexual enjoyment and more sexual discomfort among BCSs than controls, Dorval et al. [19] reported that sexually active BCSs were less satisfied with their sexual life compared to controls. In a recent Finnish study assessing health-related quality of life up to 10 years after BC treatment, BCSs reported more sexual problems than controls [38]. On the other hand, Soldera et al. [18] found no differences in sexual pleasure and sexual discomfort in sexually active BCSs compared to controls 12 years after diagnosis. This discrepancy between the Soldera et al. study and our study may be due to differences in treatment burden among BCSs, as only 66% of the BCSs in their study had received systemic adjuvant treatment including only 10% with the combination of endocrine- and chemotherapy, while the corresponding numbers in our study were 82 and 54%. Also in Soldera’s study, BCSs treated with adjuvant therapy, and especially with chemotherapy, reported worse gynecological symptoms than controls. Another study by Ganz et al. [16], reported more sexual discomfort among 6 years BCSs treated with adjuvant chemotherapy compared to those who had received tamoxifen only or no systemic therapy. To note, the studies by Soldera et al. and Ganz et al. were both conducted before the introduction of aromatase inhibitors and before extended endocrine therapy became a recommended treatment option for high-risk patients, and were thus not able to explore the impact of these treatment options as done in our study.

The present study shows a significant association between premenopausal age at diagnosis and long-term sexual health impairments in BCSs. Our findings are in line with results from another comparative study examining BCSs 3–8 years after diagnosis [23], reporting poorer sexual health in BCSs at young age (≤ 45 years) compared to both age-matched controls and BCSs who were older (≥ 55 years) at diagnosis. In our study, younger compared to older BCSs were generally diagnosed with more advanced BC, and received more intensive treatments including extended endocrine treatment.

We found no differences in sexual health between BCSs who had not received systemic adjuvant treatment and controls. The majority of these BCSs were postmenopausal at diagnosis. However, BCSs diagnosed at postmenopausal age and treated with endocrine- and chemotherapy, reported poorer sexual health than controls. David et al. [17] found worse sexual symptoms also among postmenopausal long-term BCSs not treated with chemotherapy and no longer on adjuvant endocrine therapy than among controls. The majority of these BCSs had, however, been previously treated with adjuvant endocrine therapy, supporting our finding of more sexual discomfort in postmenopausal BCSs treated with adjuvant endocrine therapy.

Thus, findings support that even though sexual activity seems not significantly affected in BCSs, systemic BC treatments have great negative impact on other aspects of sexual health among long-term BCSs, including satisfaction, enjoyment and discomfort, and mostly so in the most heavily treated.

### Strengths and limitations

At present, this is the largest study comparing sexual health in long-term BCSs with population controls. No prior study has presented data on sexual health among BCSs receiving extended endocrine treatment. The BCSs were included nationwide, and the controls were considered fairly representative for Norwegian females except for the lack of large cities and immigrant populations [30]. We could have wished for a higher response rate in SWEET, however, based on comparable studies we consider it acceptable [39, 40]. Questionnaires with established psychometric properties were used. We are not aware of other studies reporting mean scores for the domains sexual functioning and sexual enjoyment of EORTC BR23 from population-based samples. Even though this is a BC-specific questionnaire, the sexual items are relevant in the general female population.

Some methodological aspects should be considered. We chose not to exclude controls with prior and/or present cancer as this in our opinion best reflected the general population. The sexual health differences between BCSs and controls may have been even larger if women with cancer had been excluded. The group defined as premenopausal in our study probably includes some postmenopausal women, while the opposite is less likely. These aspects strengthen the finding of poorer sexual health in women diagnosed with BC at premenopausal age compared to controls.

We defined a mean score difference of ≥ 10% between groups as a clinically significant difference. Even smaller differences of 5–10% may have some clinical relevance [37], which if used would have resulted in clinically significant differences between groups for more outcomes in the analyses. BCSs in this study were treated in a period where the use of adjuvant chemotherapy peaked. Because few premenopausal BCSs were treated without systemic therapies or with endocrine therapy only, no comparative results from these treatments groups could be presented. Treatment with ovarian suppression, in addition to tamoxifen or aromatase inhibitor in premenopausal women with high-risk BC, was not standard of care in 2011 or 2012. Such treatment, which is given today [41], is associated with further reduction in sexual health [42]. BCSs > 65 years at diagnosis and BCSs with relapse or metastatic disease were not invited in the study. Thus, our results cannot be generalized to all age groups or BCSs with advanced disease.

### Conclusion

BCSs experienced more sexual health impairments 8 years after diagnosis compared to similar aged population controls. At particular high-risk of sexual health impairments are BCSs diagnosed at premenopausal age and those treated with intensive systemic adjuvant treatments. This knowledge is important, not only for BCSs, but also for the health care providers. During follow-up, attention to sexual health impairments, especially among BCSs with these risk factors, should be provided and handled according to relevant recommendations [43, 44].

## Supplementary Information

Below is the link to the electronic supplementary material.

Supplementary file1 (DOCX 18 KB)

## Data Availability

Data from SWEET is available at the National Advisory Unit for Late Effects after Cancer Treatment, Department of Oncology, Oslo University Hospital, the Radium Hospital, Oslo, Norway. Data from the HUNT study used in research projects is available upon reasonable request to the HUNT data access committee (hunt@medisin.ntnu.no).
