# Supplementary material for: Sexual health in long-term breast cancer survivors: a comparison with female population controls from the HUNT study
Source: Breast Cancer Res Treat. 2023 Jul 25;201(3):479–88. doi: 10.1007/s10549-023-07021-y (PMC10460729; doi:10.1007/s10549-023-07021-y)
Supplement: Supplementary file 1 — Supplementary file1 (DOCX 18 KB) [file 10549_2023_7021_MOESM1_ESM.docx]

| **Supplementary table 1: The complete linear regression model with sexual health measures as**  **outcome** | | | | | | |
| --- | --- | --- | --- | --- | --- | --- |
|  | **Sexual functioning** | | **Sexual enjoyment** | | **Sexual discomfort** | |
|  | Beta | 95% CI | Beta | 95% CI | Beta | 95% CI |
| Block 1  SWEET (HUNT4 ref)  Age at survey  Living with partner  Education < 13 years | -8.7  -0.5  8.7  -2.7 | -10.1, -7.3  -0.5, -0.4  7.8, 9.5  -3.5, -2.0 | -14.4  -0.4  -1.7  -3.5 | -16.4, -12.5  -0.4, -0.3  -3.0, -0.5  -4.4, -2.6 | 1.0  0.03  0.4  0.07 | 0.9, 1.1  0.02, 0.03  0.3, 0.4  0.02, 0.1 |
| Block 2  SWEET (HUNT 4 ref)  Age at survey  Living with partner  Education < 13 years  Somatic co-morbidity  -no condition  -1 condition  -≥2 conditions  Mental co-morbidity  Body mass index  Sleeping problems | -7.7  -0.5  7.9  -2.3  Ref  -0.7  -2.6  -3.8  -0.09  -3.5 | -9.2, -6.3  -0.5, -0.4  7.0, 8.8  -3.1, -1.6  -  -1.5, 0.1  -4.0, -1.3  -4.7, -3.0  -0.2, -0.02  -4.4, -2.7 | -13.1  -0.4  -2.6  -3.2  Ref  -1.0  -3.5  -3.9  0.1  -4.7 | -15.0, -11.2  -0.4, -0.3  -3.8, -1.3  -4.1,-2.4  -  -2.0, 0.03  -5.3, -1.7  -4.9, -2.8  0.05, 0.2  -5.8, -3.7 | 0.9  0.03  0.4  0.06  Ref  0.1  0.2  0.2  -0.02  0.3 | 0.8, 1.0  0.02, 0.03  0.4, 0.5  0.01, 0.1  -  0.04, 0.2  0.1, 0.3  0.2, 0.3  -0.03, -0.02  0.3, 0.4 |
| CI: confidence interval.  ¹Scale 0-100 (EORTC-BR23), ²Scale 0-100 (EORTC-BR23) among sexually active women, ³Scale 0-6 (Sexual Activity Questionnaire) among sexually active women | | | | | | |
